# Prenatal diagnosis of dent disease type I with a nonsense pathogenic variant in *CLCN5: a case study*

**DOI:** 10.1186/s12920-024-01809-7

**Published:** 2024-01-24

**Authors:** Ruijue Zhu, Mingming Zhu, Boye Wang, Enen Chen, Danlei Cai, Yinghong Yang, Yi Liang, Chuqi Su, Ding Wang, Xiaofang Sun, Linhuan Huang, Yingjun Xie

**Affiliations:** 1 Department of Obstetrics and Gynecology, Guangdong Provincial Key Laboratory of Major Obstetric Diseases, Guangdong Provincial Clinical Research Center for Obstetrics and Gynecology, Guangdong-Hong Kong-Macao Greater Bay Area Higher Education Joint Laboratory of Maternal-Fetal Medicine, The Third Affiliated Hospital of Guangzhou Medical University, No. 63 of Duobao Road, 510150 Guangzhou, China; 2 Guangzhou Municipal and Guangdong Provincial Key Laboratory of Molecular Target & Clinical Pharmacology, The NMPA and State Key Laboratory of Respiratory Disease, School of Pharmaceutical Sciences and the Fifth Affiliated Hospital, Guangzhou Medical University, 511436 Guangzhou, Guangdong China; 3 Department of Ultrasound, The First Affiliated Hospital of Sun Yat-sen University, 510100 Guangzhou, Guangdong China; 4 Department of Obstetrics and Gynecology, The First Affiliated Hospital of Sun Yat-sen University, 58 Zhong Shan Er Road, 510100 510080 Guangzhou, Guangdong China

**Keywords:** Dent disease, *CLCN5* gene pathogenic variant, Foetus, Prenatal diagnosis

## Abstract

**Introduction:**

Dent disease type I is a rare X-linked recessive renal tubular disease resulting from pathogenic variants in the *CLCN5* gene. Due to the rarity of Dent disease type I and the diversity of its phenotypes, its clinical diagnosis is complex and poses a challenge to clinicians.

**Methods:**

A foetus and a child from a 36-year-old pregnant woman with a birth history of abnormal children were enrolled in this study. Pregnant women undergo amniocentesis for prenatal diagnosis at the gestational age of 12^+ 3^ weeks. Chromosomal microarray (CMA) analysis and whole-exome sequencing (WES) were employed to investigate the chromosomal copy number and single gene variants. Literature retrieval and data analysis were performed for genotype and phenotype collection analysis.

**Results:**

No chromosomal abnormalities or CNVs were detected in the entire family through karyotype and familial CMA analyses. WES identified a nonsense pathogenic variant in *CLCN5* of the X chromosome, c.1942 C > T (exon 11, NM_000084), which was inherited from his mother, who exhibited regular clinical features.

**Conclusion:**

This study suggests that children with low-molecular-weight proteinuria and hypercalciuria should undergo prompt genetic testing to exclude Dent disease.

## Introduction

*Dent disease* is a rare X-linked recessive inherited renal tubular disease characterized by low-molecular-weight proteinuria (LMWP), hypercalciuria, renal calcium deposition, and renal insufficiency, which may eventually progress to life-threatening renal failure. Through genetic testing, Dent disease is classified into Dent disease type I (OMIM #300009) and Dent disease type II (OMIM #300555). Dent disease type I, caused by pathogenic variants in the *CLCN5* gene, accounts for approximately 60% of cases, and Dent disease type II, caused by pathogenic variants in the *OCRL1* gene, accounts for approximately 15% of cases. Dent disease with pathogenic variants in both the *CLCN5* and *OCRL1* genes has also been reported [1], but the causative genes in 25% of Dent disease patients are still unknown and are collectively referred to as Dent disease type III [2]. To date, 266 different *CLCN5* pathogenic variants have been described [3]. Most reported pathogenic variants are missense or frameshift variants, followed by nonsense pathogenic variants.

The *CLCN5* gene is located on chromosome Xp11.23 with a coding region of 2,238 bp [4]. The protein encoded by the *CLCN5* gene is CLC-5, which belongs to the CLC channel and ion exchange family and is a Cl^−^/H^+^ reverse transporter protein [5]. CLC-5 is involved in protein uptake by the brush border of proximal tubular cells (PTCs) and early endosomal acidification [3]. It also plays a vital role in the endocytosis pathway of the proximal tubule [6]. In the kidney, CLC-5 is expressed on the proximal tubule, the thick segment of the ascending branch of the medulla, and the collecting duct; CLC-5 is also expressed in podocytes [7], which suggests that CLC-5 may also play a role in glomerular function. The chloride concentration maintained by CLC-5 is essential for the reabsorption of low-molecular-weight protein and albumin by the proximal tubule [8]. Patients with Dent disease type I may also exhibit decreased expression of megalin and cubilin-amnionless complexes in PTCs, which may also contribute to the development of proteinuria [3]. As shown in public databases, *CLCN5* pathogenic variants also cause hypophosphatemic rickets (OMIM:#300554), renal stone type I (OMIM:#310468) and low-molecular-weight proteinuria with hypercalciuric renal calcinosis (OMIM: #308990).

Here, we analysed the clinical data and outcomes of a child with Dent disease type I and a foetus suspected of having Dent disease type I. To discuss the prenatal diagnosis of Dent disease and improve clinicians’ understanding of the disease, previously reported cases were reviewed.

## Patients and methods

### Subjects

**Fig. 1:**
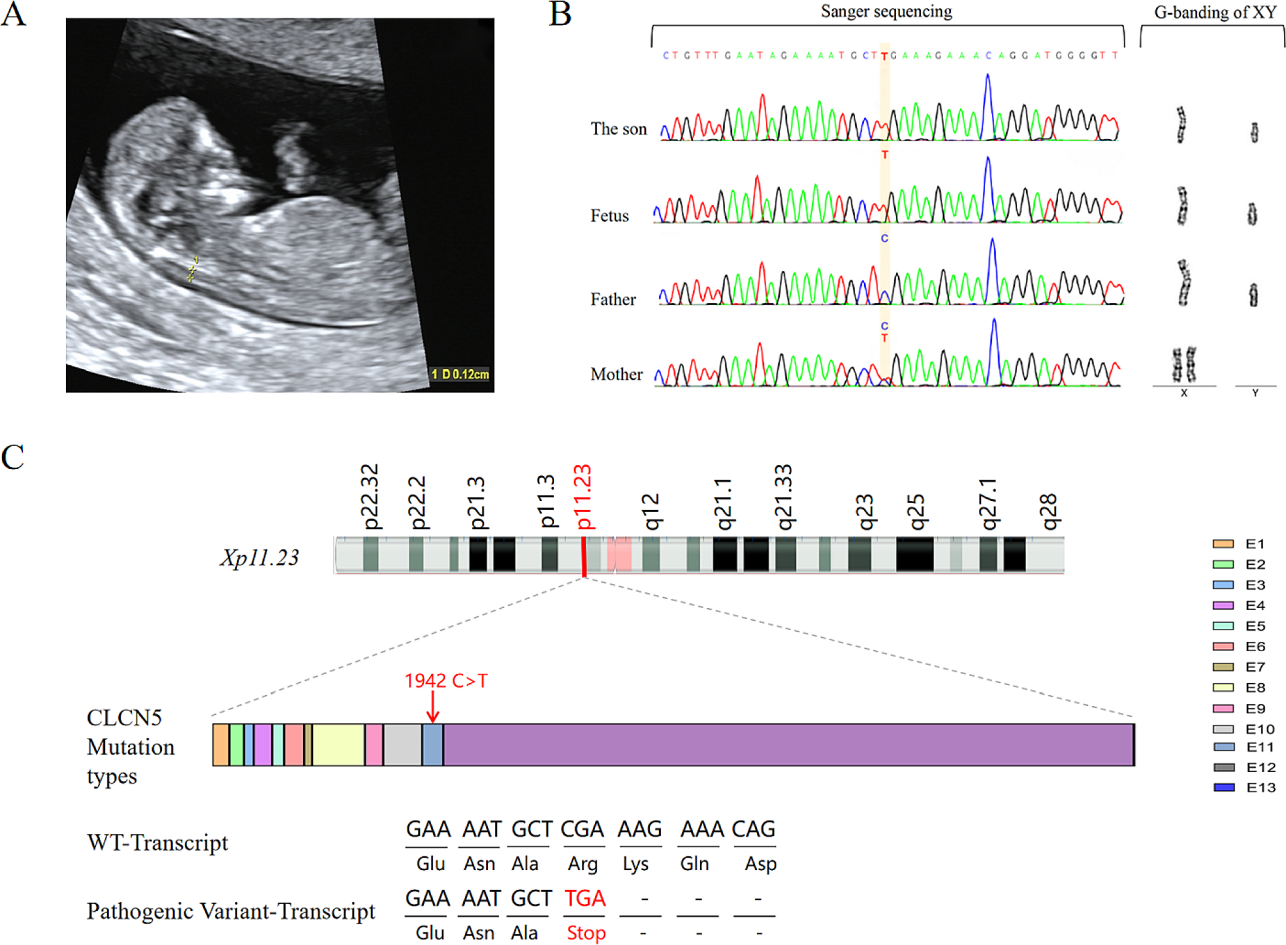
(**A**) Ultrasound scan of a foetus in early pregnancy. (**B**) Sequence analysis of the CLCN5 gene in the foetus, son and parents confirmed the novel c.1942 C > T variant in exon 11. (**C**) Schematic representation of the CLCN5 gene structure reflecting the c.1942 C > T (exon 11, NM_000084.5) variant described in this paper. The pathogenic variant changes the arginine (CGA) encoded in exon 10 to a stop codon (p.R648*) (TGA), resulting in the loss of 100 amino acids

A 36-year-old pregnant woman, gravida 2, para 1, came to the First Affiliated Hospital of Sun Yat-sen University because of a birth history of a son with Dent disease. Ultrasound *B*-mode showed that the crown-rump length of the foetus at 11^+ 6^ weeks of gestation was 51 mm, with no abnormality of soft ultrasound indicators in early pregnancy and small uterine fibroids (intermural 12 mm×7 mm) (Fig. 1A).

The first child of the pregnant woman, a 6-year-old son, was a term and natural-labour male with a birth weight of 3 kg and without a history of choking-rescue. Foamy urine was observed when he was 5^+^ years old, as well as urological abnormalities, LMWP, hypercalciuria, renal tubular abnormalities, hypokalaemia, and secondary aldosteronism. The son and his parents underwent whole-exome sequencing (WES). The findings revealed a hemizygous pathogenic variant in the *CLCN5* gene of the X chromosome in both the foetus and the son. Specifically, the pathogenic variant involved a C-to-T substitution at position 1942, designated c.1942 C > T (exon 11, NM_000084) (Fig. 1B).

The parents were healthy with no personal or familial history of depression; the mother was healthy during pregnancy and had no prenatal complications. Ethical approval was obtained for this study from the Institutional Review Board of The Third Affiliated Hospital of Guangzhou Medical University (NO: 2023062). All data were collected with the patient’s informed consent.

### Karyotype analysis

Approximately 2 ~ 3 ml of peripheral blood was collected from the patient and the parents for karyotype analysis. After staining with Giemsa stain, twenty karyotypes were counted, and five karyotypes were analysed, which were consistent with the International System for Human Cytogenetic Nomenclature (ISCN) 2020.

### Chromosomal microarray (CMA) analysis

With the consent of the pregnant woman and her family, amniocentesis was performed at 12^+ 3^ weeks of gestation. The villi were examined by CMA analysis. CMA analysis of the uncultured amniotic fluid was performed using an Affymetrix cyto HD Array (Affymetrix, High Wycombe, UK). DNA was amplified, labelled, and hybridized to a CytoScan HD array platform according to the manufacturer’s protocol. The array was explicitly designed for cytogenetics research and included more than two million markers across the genome, including SNP probes and probes to detect copy number variations (Cytoarrays). CEL files, obtained by scanning the CytoScan arrays, were analysed with Chromosome Analysis Suite software (Affymetrix) using annotations of genome version GRCH37 [9].

### Whole-exome sequencing (WES)

WES was performed using complete exon high-throughput sequencing. Subsequently, data were analysed using the Verita Trekker® pathogenic variant detection system and Enliven® pathogenic variant annotation and interpretation system independently developed by Berry Genetics. Pathogenic variants were assessed under the protocol issued by ACMG [10]. The Human Gene Mutation Database (HGMD) was used to screen pathogenic variants reported in published studies.

### Sanger sequencing

To validate the DNA sequence variants in the *CLCN5* gene identified through WES, the target sites were amplified using forward (5’-accctggcaatggatgtgatgaa-3’) and reverse (5’-cggcagtgcctggttacacaca-3’) primers, which were predicted to generate a 409 bp product. The PCR conditions were as follows: degeneration at 94 °C for 3 min; 35 cycles of 94 °C for 30 s, 58 °C for 30 s, and 72 °C for 30 s; and extension at 72 °C for 5 min. PCR products were purified and sequenced. Gene sequencing was performed using ABI 3730XL.

### In silico analysis

The National Center for Biotechnology Information Database (https://www.ncbi.nlm.nih.gov/) was used to collect 11 different species gene sequences, and the gene sequences were imported into DNAMAN Version 6 software for multiple sequence alignment.

### Literature retrieval and data extraction

To compare the clinical phenotype of this patient with that of other reported patients with the same pathogenic variant, “(Dent disease) and (*CLCN5*)” were used as the formula for literature retrieval in the PubMed database (https://pubmed.ncbi.nlm.nih.gov/). Studies with the same pathogenic variant as this case were screened without the restriction of search time, and patient country, publication year, sex, age of onset, age of diagnosis, gene pathogenic variant site, amino acid change, pathogenic variant source, clinical features, renal pathology and so on were extracted.

## Results

### Karyotype analysis and CMA analysis

The CMA results showed no copy number variation or chromosome imbalance, and no abnormalities of genome imbalance, such as microdeletion and microduplication, were detected.

### WES

WES detected a hemizygous pathogenic variant in the *CLCN5* gene of the foetal X chromosome, c.1942 C > T (exon 11, NM_000084.5). The pathogenic variant changes the encoded arginine into a stop codon, p.R648*, which stops translation and causes the loss of 100 amino acids (Fig. 1C). According to the American College of Medical Genetics and Genomics (ACMG) guidelines [11] and the recommendation of the ClinGen Sequence Variant Interpretation (SVI) panel [12], c.1942 C > T was suggested to be a pathogenic variant (PVS1 + PM2 + PP4 + PS4_Supporting). In addition, the known variation was evaluated as P in the ClinVar database and DM in the HGMD. No pathogenic variant of *the CLCN5* gene was detected in the father, and a heterozygous pathogenic variant of the *CLCN5* gene was detected in the mother. In addition, the same hemizygous pathogenic variant of the *CLCN5* gene was confirmed in the parents’ first child. The pregnant woman underwent genetic counselling and returned to the local area to induce labour.

### Sanger sequencing

Sanger sequencing showed that the son and the foetus had a hemizygous pathogenic variant in the *CLCN5* gene of the X chromosome, c.1942 C > T (exon 11, NM_000084) (Fig. 1B).

### In silico analysis

**Fig. 2:**
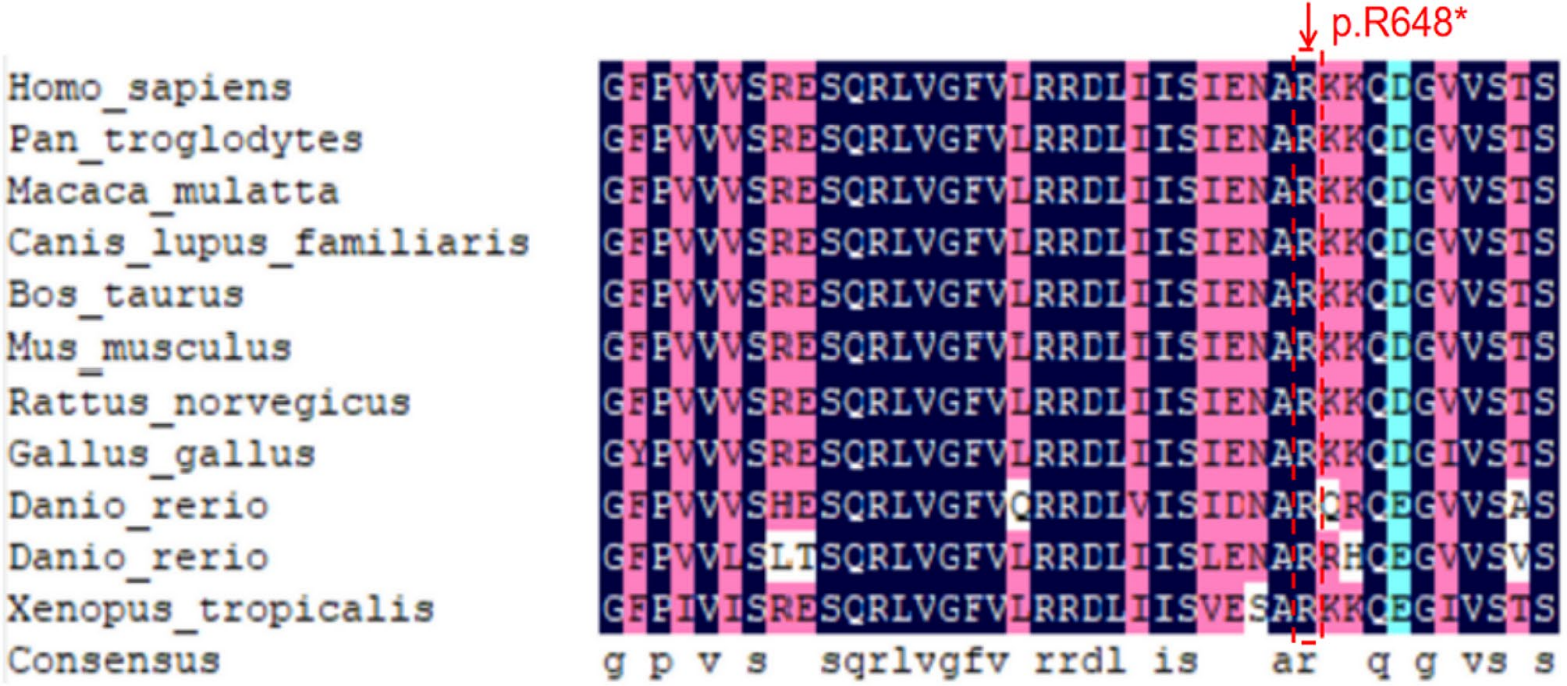
Multiple comparisons were made between human CLCN5 (NP_597680.2) and selected homologues

Multiple comparisons were made between human *CLCN5* (NP_597680.2) and selected homologues, such as pan troglodytes (XP_003317509.2), *Macaca mulatta* (XP_001083186.2), Canis lupus familiaris (XP_005641410.1), *Bos taurus* (XP_005228237.1), *Mus musculus* (NP_001230691.1), *Rattus norvegicus* (NP_058802.1), Gallus gallus (XP_004940778.1), *Danio rerio* (XP_001920783.2), *Danio rerio* (XP_685762.2) and *Xenopus tropicalis* (NP_001039210.1). Conservation analysis showed that no highly conserved amino acid region at the p.R648X site was affected (Fig. 2).

### Literature retrieval and data analysis

Lloyd et al. first reported this pathogenic variant, and five studies in total reported this pathogenic variant [13–17]. There were 7 cases, all male, diagnosed between 0.5 and 14 years old. Except for 2 cases with LMWP, there was heterogeneity in the clinical features of the different cases (Tables 1 and 2). However, in this case, the son had clear foamy urine, renal tubular abnormalities, hypokalaemia and secondary hyperaldosteronism but no renal calcinosis or rickets.

**Table 1:** The basic situation of children with Dent disease with the same pathogenic variant that has been reported in the literature

Number	Authors	Patient’s country	Year of publication	Gender	Age of onset (years)	Age at diagnosis (years)	Gene mutation site	Amino acid variation	Source of mutation	clinical characteristics	Kidney pathology	other
1	Lloyd et al.	Italy	1997	M	/	3	CGA→TGA	p.R648X	Family	LMWP, Nephrocalcinosis, Nephrocalcinosis, Kidney failure, Rickets	/	/
2	Lloyd et al.	Italy	1997	M	/	0.5	CGA→TGA	p.R648X	Family	LMWP, Hypercalciuria, Nephrocalcinosis, Kidney failure, Rickets	/	/
3	Igarashi et al.	Japan	1998	M	/	14	CGA→TGA	p.R648X	Mother	LMWP, Nephrocalcinosis, Nephrocalcinosis, UOB	sclerosis	Urinary calcium/creatinine: 6
4	Igarashi et al.	Japan	1998	M	/	6	CGA→TGA	p.R648X	Mother	LMWP, Nephrocalcinosis, Nephrocalcinosis, UOB	Glomerulosclerosis	Urinary calcium/creatinine: 17
5	Frishberg et al.	Israel	2009	M	7	9	/	p.R648X	Mother	Asymptomatic NRP, Mild nephrocalcinosis	FSGS and (or) Global glomerulosclerosis, Mild mesangial hyperplasia	Prenatal ultrasound shows bilateral hydronephrosis
6	Frishberg et al.	Israel	2009	M	11	11	/	p. R648X	Mother	Asymptomatic NRP, Mild nephrocalcinosis	FSGS and (or) Global glomerulosclerosis	/
7	Bhardwaj et al.	India	2016	M	1	14	c.1942 C > T	p. R648X	/	LMWP, Hypercalciuria, Rickets, Night blindness, Amino acid urine	/	/

Abbreviation: M: Male; LMWP: Low-molecular weight proteinuria; UOB: Urine occult blood; NRP: Nephrotic-range proteinuria; FSGS: Focal segmental glomerulosclerosis

**Table 2:** Clinical phenotype of patients with CLCN5: p.R648*

Sex (male, female)	100% male
	0% female (7: 0)
clinical characteristics	
LMWP	71.4% (5/7)
Hypercalciuria	71.4% (5/7)
Nephrocalcinosis	85.7% (6/7)
Asymptomatic NRP	28.6% (2/7)
Rickets	42.9% (3/7)
UOB	28.6% (2/7)
Night blindness	14.3% (1/7)
Amino acid urine	14.3% (1/7)
Renal failure	28.6% (2/7)

Abbreviation: LMWP: Low-molecular-weight proteinuria; NRP:Nephrotic-range proteinuria;UOB:Urine occult blood

## Discussion

Dent and Friedman first reported two cases of Dent disease in 1964 [18]. Although cases of Dent disease have been reported at home and abroad, the current understanding of Dent disease is still insufficient. Due to the inadequacy of examinations and the heterogeneity of clinical features, the disease is prone to misdiagnosis and mismanagement, and the incidence of Dent disease is unknown. In recent years, the development of diagnostic methods such as genetic testing has helped diagnose the disease. Mutilation disease is a congenital genetic disease, and it remains incurable. Only symptomatic support, including reduction of hypercalciuria, prevention of nephrocalcinosis and nephrolithiasis, and delay in the progression of chronic kidney disease, can be provided. Therefore, there need to be more effective treatment methods [19–24].

There are no reports on the prenatal diagnosis of Dent disease type I with a nonsense pathogenic variant of the *CLCN5* gene. The pregnant woman came to our hospital because of a “Dent disease childbearing history.” We performed CMA analysis and WES on the foetal villi. The results showed a hemizygous pathogenic variant in the CLCN5 gene of the foetal X chromosome, c.1942 C > T (p.R648*). This variant was a nonsense pathogenic variant that resulted in the truncation of the *CLCN5*-encoded CLC-5 channel protein. Our study represents the first case of prenatal diagnosis for Dent disease type I with a nonsense pathogenic variant of the *CLCN5* gene. However, the phenotypes of LMWP or hypercalciuria were unable to be determined throughout the foetal period. Furthermore, the disease does not seem to cause any abnormality in the foetus that can be detected by ultrasound.

Cases in the literature with the same pathogenic variant as that in this study were summarized and analysed (Tables 1 and 2). The analysis of these 7 cases revealed heterogeneity in clinical features, among which LMWP was found in 71.4% (5/7), hypercalciuria was found in 71.4% (5/7), and renal calcinosis was found in 85.7% (6/7). The incidences of urine occult blood (UOB), nephropathy-range proteinuria (NRP), amino acid urine and night blindness are low, as shown in Table 2. Renal pathology analysis showed that the pathogenic variant mainly caused glomerulosclerosis, as shown in Table 1. In an extensive study of 30 kidney biopsies, focal global glomerular sclerosis and tubulointerstitial fibrosis were the most common pathologic types, found in 83% and 60% of cases, respectively [25]. In this case, LMWP and hypercalciuria were also present. Therefore, LMWP and hypercalciuria may be the main symptoms of this pathogenic variant. Furthermore, the diverse clinical phenotypes may be related to geographical and racial factors.

The core clinical symptom of Dent disease is LMWP, characterized by renal tubular proteinuria. LMWP is found in 99% of male Dent patients, including α1-microglobulin, β2-microglobulin, retinol-binding protein, Clara cell protein, and vitamin D binding protein [26]. Moreover, oedema, hyperlipidaemia, and albumin reduction are less likely, which can differentiate Dent disease from nephrotic syndrome and Fanconi syndrome. Van Berkel et al. reported that glomerulosclerosis and tubulointerstitial fibrosis were present in childhood Dent patients even with normal renal function [7]. Therefore, quantitative and qualitative measures should be considered for children with proteinuria, and renal tubular proteinuria should not be ignored. If LMWP is identified by urine protein electrophoresis, the presence of other clinical symptoms, such as hypercalciuria, haematuria, etc., should be considered to determine whether to conduct genetic testing to exclude Dent disease.

In conclusion, the clinical manifestations of Dent disease are diverse, and there are no known biomarkers for use in prenatal diagnosis, which increases its difficulty. A child with a birth history of LMWP and hypercalciuria should undergo WES if necessary to confirm Dent disease.

## Data Availability

Whole-exome sequencing data of variable sites are available in the NCBI SRA under the accession number PRJNA 1022045 (https://www.ncbi.nlm.nih.gov/sra/PRJNA1022045).
